# A rare case of congenital heart disease with ambiguous genitalia

**DOI:** 10.4103/0971-6866.73414

**Published:** 2010

**Authors:** Kusuma Lingaiah, Bharath A. Parshwanath, Savitha R. Mysore, Balasundaram Krishnamurthy, Nallur B. Ramachandra

**Affiliations:** Department of Studies in Zoology, Human Genetics Laboratory, University of Mysore, Manasagangothri, Mysore - 570 006; 1 Department of Pediatrics, Cheluvamba Hospital, Mysore Medical College, Mysore, Karnataka, India

**Keywords:** Ambiguous genitalia, chromosome, congenital heart disease, pseudohermaphroditism

## Abstract

Birth defects have become the important cause of mortality and morbidity in the perinatal period. Congenital heart disease (CHD) is the most common birth defect which includes the varying forms of cardiac abnormalities and occurs with an incidence of 1 per 100 live births. In most of the cases, CHD is an isolated malformation, but about 33% have associated anomalies. Ambiguous genitalia are one such rare anomaly that is associated with CHD among other genital abnormalities. The possible causes for this association could be pseudohermaphroditism, which in turn, may be due to congenital adrenal hyperplasia. The government of any country should consider providing for its people a free prenatal diagnosis for susceptible disorders.

## Introduction

Developmental heart disorders are the most common of all human birth defects and occur in nearly 1% of the population.[1] Congenital malformations of the heart and great vessels are among the most frequent of all congenital anomalies detected during 1^st^ year of life.[2] They constitute one of the major causes of infant mortality and morbidity in childhood and in later adult life. Extracardiac anomalies occur in 15–45% of cases with Congenital Heart Disease (CHD).[3,4] Some of them are: craniofacial anomalies, genitourinary, musculoskeletal, respiratory, gastrointestinal, central nervous systems and spleen anomalies. Here, we report a very rare case of CHD associated with ambiguous genitalia.

## Case Report

**Figure 1:**
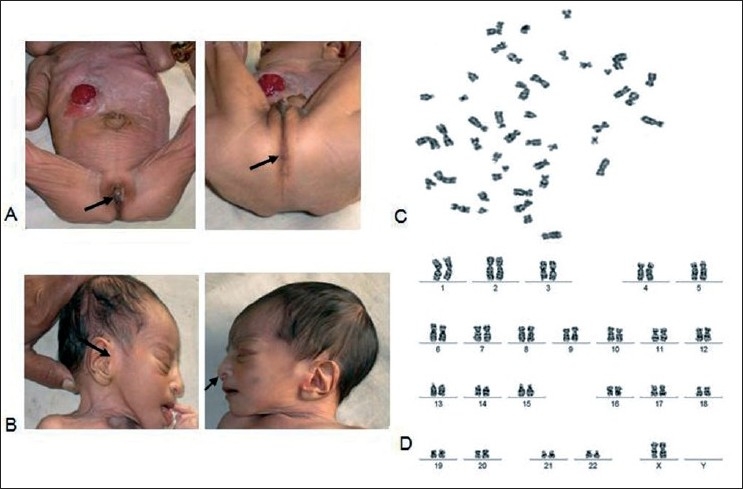
(A) Clinical features of ambiguous genitalia, closure of anal opening at birth, umbilical hernia; (B) features showing ear pits on both ears, down-slanting nasal tip; (C) metaphase plate; (D) karyotype of the patient with ambiguous genitalia.

A 1-day old infant, born at the Cheluvamba Hospital, Mysore, had symptoms of CHD associated with ambiguous genitalia and was the subject for the present investigation. Ambiguous genitalia is a condition where there is an abnormal development of the genital organ, which creates a question about the child’s gender. Clinical investigations revealed that the child had absence of anal opening at birth, ambiguous genitalia, umbilical hernia, periauricular pits, down-slanting nasal tip, depressed nasal bridge, bilateral hydronephrosis [Figure 1A and B, Table 1] and a possible CHD. The ultrasonography of the internal organs revealed that the proband had both the ovaries and uterus, indicating the presence of an internal female reproductive organ. Echocardiographic examinations revealed a ventricular septal defect (VSD) along with atrial septal defect (ASD) as CHD. Clinical evaluation of the features observed in the proband also indicated that the patient had a Kabuki-like syndrome [Table 1]. The proband was the second child of non-consanguineous parents. At the time of conception, the age of the mother and the father were 24 and 28 years, respectively. The birth weight of the proband was 2600 g. The pedigree analysis showed no family history of the CHD or other developmental disorders.

**Table 1:** Clinical features of Kabuki syndrome observed in the proband

Clinical Features of Kabuki syndrome	Features observed in the proband
Long palpebral fissures	-
Lower palperbral eversion	-
Arched eyebrows	-
Long eyelashes	-
Blue sclera	-
Ptosis	-
Depressed nasal tip	+
Cleft lip/ palate/arched palate	-
Dysmorphic ears	+
Preauricular pits	+
Abnormal dentition	-
Respiratory infection	-
Cardiovascular malformation	+
Microcephaly	-
Seizures	-
Diaphragm hernias/eventration	+
Malrotation of intestines	-
Abnormalities with anus	+
Abnormal skin(hyperelastic)	+
Prominent fingertip pads	-
Unusual dermatoglyphic pattern	-
Skeletal abnormalities	-
Mental retardation	-
Postnatal growth deficiency	-
Bilateral hydronephrosis	+

Chromosomal analysis of the proband using standard protocol of Seabright[5] with certain modifications revealed the normal female complement with 2*n* = 46 (44 + XX) [Figure 1c and d]. The study was approved by the ethical committee of University of Mysore with Institutional Human Ethical Committee (IHEC) (numbered D33/2008–09). Blood sample was collected after obtaining written consent from the patient’s parents. A total of 100 G-banded metaphases were analyzed and karyotyped according to the International System for Human Cytogenetic Nomenclature[10], using the Leica DMRA2 trinocular research microscope.

## Discussion

Review of literature reveals that ambiguous genitalia are rare extracardiac anomalies. Ambiguous genitalia occur with an incidence of 1 per 50,000–70,000.[6] The causes of ambiguous genitalia are many; some of them are true hermaphroditism, gonadal dysgenesis, chromosomal mosaics and pseudohermaphroditism. Taking into cognizance all the investigations conducted in the present study, it can be said that one of the possibilities is that the proband is a pseudohermaphrodite which could be due to congenital adrenal hyperplasia, as reported by earlier workers.[7,8] The frequency of this disorder in the United States and Europe is 1 in 5000 and 1 in 15,000, respectively. This common disorder is inherited as an autosomal recessive trait.[7] The deficiency of enzyme17-hydroxy steroids causes ambiguous genitalia. The metabolic abnormality results in deficient cortisol production, which increases the level of ACTH release with further steroid stimulation and an elevation of circulating androgens. However, the biochemical assay of 17-hydroxy steroid was not carried out for the patient due to his death.

On the other hand, the proband also had features of Kabuki-like syndrome which is a multiple congenital anomaly characterized by specific facial features, mild to moderate mental retardation, postnatal growth delay, skeletal abnormalities, and unusual dermatoglyphic patterns with prominent fingertip pads with an estimated incidence of 1 in 32,000 newborns.[9] However, we could observe only few of these features in the proband.

Therefore, the causes of ambiguous genitalia with CHD could be any of the above discussed possible factors. Unfortunately, further study of the proband was not possible due to its death during these investigations. However, with the available information, one can surmise that this rare case of CHD with ambiguous genitalia having normal female chromosome complement could be due to congenital adrenal hyperplasia. To our knowledge, this is a maiden report of CHD with ambiguous genitalia.

Interestingly, these conditions can be identified by prenatal screening and can be treated or corrected through steroid administration to the mother from the 10^th^ week, which may prevent ambiguous genitalia in female fetuses.[7] To overcome the ambiguity and also to rectify the defect, a surgical intervention can be used. This anomaly could have been detected if prenatal diagnosis was carried out by ultrasonography. Therefore, a free and compulsory prenatal diagnosis of at least suspected family should be done by the intervention of the public sector of any country, so that we can prevent and avoid abnormal birth.
